# Exercise training and dynamic cerebral autoregulation: Is adaptation possible?

**DOI:** 10.1113/EP093703

**Published:** 2026-03-04

**Authors:** Patrice Brassard

**Affiliations:** ^1^ Department of Kinesiology, Faculty of Medicine Université Laval Québec Canada; ^2^ Research Center of the Institut Universitaire de Cardiologie et de Pneumologie de Québec Québec Canada

**Keywords:** dynamic cerebral autoregulation, exercise training, transfer function analysis

1

Endurance and resistance exercise generally promote positive adaptations for brain and cognitive health. Focusing specifically on the cerebrovascular function, habitual endurance exercise attenuates the age‐related reduction in cerebral blood velocity and both endurance and resistance training improve cerebrovascular reactivity to carbon dioxide. However, despite these benefits, some evidence suggests that habitual endurance or resistance exercise, and/or higher cardiorespiratory fitness, do not improve – and may even worsen – dynamic cerebral autoregulation (dCA), which reflects the ability of the cerebrovasculature to respond to transient changes in arterial blood pressure (ABP) (discussed in Labrecque et al., 2017). Yet, current findings from longitudinal studies remain limited, and dCA is often characterized using a single metric. Because different physiological stressors (e.g., spontaneous vs. forced ABP oscillation) can uniquely engage and challenge the regulatory mechanisms underlying dCA, and because most dCA metrics appear unrelated to one another, a multimetrics approach is necessary to enhance interpretation and understanding of this complex physiological process.

In this issue of *Experimental Physiology*, Thomas et al. (2026) used a randomized cross‐over experimental design to compare the effects of 12‐week intensity‐matched (based on heart rate) supervised resistance and endurance exercise interventions on dCA between healthy males and females. dCA was assessed using transfer function analysis (TFA) during both spontaneous and forced ABP oscillations induced by repeated squat–stands. The authors found that females exhibited reduced dCA and higher middle cerebral artery blood velocity at baseline in comparison with males. Furthermore, 3 months of resistance exercise training improved dCA – an effect observed in females but not males – during spontaneous and forced ABP oscillations. In contrast, 3 months of endurance exercise training did not alter dCA. These findings support prior cross‐sectional and limited longitudinal evidence indicating that endurance exercise training does not improve dCA. Although earlier cross‐sectional studies – predominantly in males – reported unchanged dCA among resistance‐trained individuals, the results of Thomas et al. (2026) suggest beneficial cerebrovascular adaptations to resistance exercise training in young healthy females.

This improvement in dCA following resistance exercise training is surprising, given no solid evidence currently supports the idea that long‐term exercise training reliably or robustly enhances dCA. Indeed, cross‐sectional studies showed no differences in dCA between older master athletes and age‐matched sedentary controls, and between trained and sedentary individuals (discussed in Labrecque et al., 2017). In addition, we also previously reported impaired dCA in young endurance‐trained males compared to age‐matched controls (Labrecque et al., 2017), which was further altered in these athletes after 6 weeks of high‐intensity interval training to exhaustion (Drapeau et al., 2019).

This raises a fundamental question: Can we meaningfully enhance dCA over time? To my knowledge, and aside from the current findings from Thomas et al., no intervention – including exercise training – has yet been shown in humans to reliably or consistently improve dCA longitudinally. Why might dCA exhibit such limited plasticity? One possibility is that it relies on rapid, reflexive mechanisms optimized for stability rather than adaptation. Another is that measurement variability may exceed any plausible training‐induced changes. Biological factors – such as biological sex, age or hormonal status – may dominate inter‐individual variability. Although dCA can be acutely influenced by physiological stressors, these short‐term effects do not appear to translate into lasting or trainable adaptations. Collectively, these observations support the view that dCA is fundamentally a ‘stability‐first’ system rather than an adaptable one.

This makes the findings of Thomas et al. (2026) both intriguing and exciting. As the authors note, the observed improvement in females might simply reflect their lower baseline dCA, giving them more room for improvement. However, just as there is little evidence that exercise training improves dCA in individuals with ‘normal’ autoregulatory capacity, there is also no clear scientific evidence that it improves dCA from an altered state – either in healthy individual or clinical populations. Also, it is worth noting that end‐tidal carbon dioxide ($P_{\mathrm{ETC}O_{2}}$) was lower at baseline in females compared with males. Although $P_{\mathrm{ETC}O_{2}}$ remained stable during all dCA assessments (beginning of manoeuvre to end) and across all study time points (pre and post each intervention), it is reasonable to assume that $P_{\mathrm{ETC}O_{2}}$ was lower at the beginning of each squat–stand manoeuvre in females based on baseline data. Since hypocapnia is known to improve dCA, it cannot be excluded that lower $P_{\mathrm{ETC}O_{2}}$ contributed – at least in part – to the observed improvement in dCA following resistance exercise training.

Alternatively, if resistance exercise training does not enhance the active component of the cerebral pressure–flow relationship, what then accounts for the apparent improvement in dCA in females when assessed using TFA metrics? The authors discuss the potential influence of vascular compliance – the passive ability of blood vessels to stretch and accommodate changes in blood volume or pressure to ensure continuous blood flow to smaller downstream vessels – on their findings. This is plausible, as previous studies using the Windkessel model indicate that capacitive blood flow is a key factor in cerebral haemodynamics and is closely linked to several TFA‐derived dCA metrics. These studies also show that Windkessel properties play a crucial role in shaping cerebral blood flow responses that are traditionally attributed to cerebral autoregulation (Tzeng et al., 2014). Animal studies have shown that young female mice possess more compliant cerebral arteries (Choi et al., 2025). While resistance exercise training appears to reduce central arterial compliance, its effects on cerebrovascular compliance remain unclear. As Windkessel models of the cerebral circulation suggest that reductions in cerebrovascular compliance lower TFA gain, the changes in TFA metrics observed in females following resistance exercise training – interpreted as improved dCA – may instead reflect a reduction in cerebrovascular compliance resulting from repeated exposure to large, frequent ABP fluctuations. Nonetheless, further research will be necessary to examine whether baseline cerebrovascular compliance predicts adaptation magnitude following resistance exercise training.

Middle‐aged endurance athletes exhibit lower cerebrovascular impedance (the functional opposite of cerebrovascular compliance) compared with their sedentary peers, and long‐term endurance exercise training can reduce cerebrovascular impedance in older adults and patients with amnestic mild cognitive impairment. However, evidence for meaningful endurance‐training‐induced changes in cerebrovascular structure and impedance or compliance in younger adults is less consistent. It is therefore plausible that the endurance exercise training stimulus applied in this study was insufficient to alter cerebrovascular compliance in young, healthy male and female participants.

I congratulate the authors on this very interesting study, and I hope these surprising findings will stimulate further investigation into the potential for resistance exercise training to improve dCA, or more broadly, modulate cerebrovascular responses to ABP changes, using a comprehensive multimetrics approach.

## AUTHOR CONTRIBUTIONS

Sole author.

## CONFLICT OF INTEREST

The author declares he has no conflicts of interest.

## FUNDING INFORMATION

No funding was received for this work.
